# The therapeutic efficacy of azithromycin and nitazoxanide in the acute pig model of *Cryptosporidium hominis*

**DOI:** 10.1371/journal.pone.0185906

**Published:** 2017-10-03

**Authors:** Sangun Lee, Melanie Harwood, Don Girouard, Marvin J. Meyers, Mary A. Campbell, Gillian Beamer, Saul Tzipori

**Affiliations:** 1 Department of Infectious Disease and Global Health, Cummings School of Veterinary Medicine at Tufts University, North Grafton, Massachusetts, United States of America; 2 Center for World Health and Medicine, Saint Louis University School of Medicine, St. Louis, Missouri, United States of America; UAMS/ACHRI/ACNC, UNITED STATES

## Abstract

Recent reports highlighting the global significance of cryptosporidiosis among children, have renewed efforts to develop control measures. We have optimized the gnotobiotic piglet model of acute diarrhea to evaluate azithromycin (AZR), nitazoxanide (NTZ), or treatment with both against *Cryptosporidium hominis*, the species responsible for most human cases. Piglets, animals reproducibly clinically susceptible to *C*. *hominis*, when inoculated with 10^6^ oocysts, developed acute diarrhea with oocyst excretion in feces within 3 days. Ten day-treatment with recommended doses for children, commencing at onset of diarrhea, showed that treatment with AZR or NTZ relieved symptoms early in the treatment compared with untreated animals. Piglets treated with AZR exhibited no reduction of oocyst excretion whereas treatment with NTZ significantly reduced oocyst shedding early, increasing however after 5 days. While treatment with AZR+NTZ led to considerable symptomatic improvement, it had a modest effect on reducing mucosal injury, and did not completely eliminate oocyst excretion. Doubling the dose of AZR and/or NTZ did not improve the clinical outcome, confirming clinical observations that NTZ is only partially effective in reducing duration of diarrhea in children. This investigation confirms the gnotobiotic piglet as a useful tool for drug evaluation for the treatment of cryptosporidiosis in children.

## Introduction

The genus *Cryptosporidium* includes enteric protozoan species which have a worldwide distribution and are found in all classes of vertebrates. *C*. *parvum* and *C*. *hominis* are the most common cause of diarrheal illness in humans. Investigations in sub-Saharan Africa [1, 2] concerning cryptosporidiosis have reported that >70% of cases in children are attributed to *C*. *hominis* and ~20% to *C*. *parvum*, of which >90 are classified as anthroponotic, a unique subspecies that appears to perpetuate within the human populations, and genetically and biologically are different from the bovine *C*. *parvum* species. The occurrence of mixed infections as well as with other *Cryptosporidium* species have been reported infrequently in humans [1, 2].

Cryptosporidiosis in young children under the age of 2 years in developing countries is a major cause of diarrhea and part of the vicious cycle of enteric infection, chronic diarrhea and malnutrition. Infections are also associated with long term sequelae including increased risk of diarrhea from other pathogens, stunted growth, and cognitive impairment [3, 4]. Investigations in sub-Saharan Africa and south Asia, ranked *Cryptosporidium* as the second leading pathogen causing life-threatening diarrhea with an estimated 7.6 million cases and 202,000 deaths annually [2, 5].

While diarrhea in healthy individuals due to *Cryptosporidium* is self-limited and lasts 1–2 weeks, infection can become chronic and life-threatening in young children with malnutrition and immunocompromised individuals [6]. Infections among HIV-infected individuals may also become extra-intestinal, spreading to other sites including the gall bladder, biliary tract, pancreas, and pulmonary system [6, 7].

The current therapeutic options for cryptosporidiosis are limited and only partially effective. Nitazoxanide (NTZ) and azithromycin (AZR) are among the most widely used drugs for treating cryptosporidiosis in human. NTZ, a nitrothiazole benzamide compound, has been reported to be effective against a wide variety of protozoa and helminths [8, 9]. Since NTZ has been approved for treatment of *Cryptosporidium* in immunocompetent children and adults, the efficacy of NTZ has been evaluated in several experimental settings, and in clinical trials [10, 11]. Although NTZ shortens the duration of diarrhea and parasite shedding in immunocompetent adults, its efficacy is limited in children and is ineffective in immunocompromised patients [12]. In calves experimentally infected with *C*. *parvum*, the efficacy of NTZ is mixed: Schnyder et al [13] reported a limited symptomatic reduction and minimal impact on oocyst excretion, while Ollivett et al. [14] reported a slightly better outcome in terms of shedding and impact on fecal consistency.

AZR is a macrolide antibiotic useful for treatment of numerous bacterial infections, and was reported to be effective in steroid-immunosuppressed rats [15]. It was evaluated as an anti-*Cryptosporidium* agent in humans and animals [16–23]. AZR treatment effectively cured a child aged 11 years infected with *Cryptosporidium* by reducing fecal oocysts [18] and lead to prompt clinical improvement in two children with cancer and severe *Cryptosporidium*-associated diarrhea [16]. In AIDS patients, AZR treatment also improved diarrhea but failed to eradicate the infection [19]. In other reports, however, AZR appears ineffective in AIDS patients with cryptosporidial diarrhea. These reports provide a suggestion and support further study, but that no actual conclusions can be drawn about the efficacy of azithromycin for treatment of cryptosporidiosis in AIDS patients. Finally, combining these two drugs is attractive but has undergone limited testing in vivo in humans or animal models. In vitro, the combination of NTZ and AZR was highly effective in inhibiting *C*. *parvum* growth compared to NTZ alone [24]. However, a combined AZR+NTZ treatment was ineffective in patients with AIDS and disseminated cryptosporidiosis [23].

In contrast to *C*. *hominis* which is host-restricted to humans, *C*. *parvum* infects all mammalian species including humans, and laboratory and domestic animals. Consequently laboratory and field investigations over the last 40 years were focused on *C*. *parvum*. The gnotobiotic (GB) piglet acute diarrhea model was shown to be susceptible to infection with *C*. *hominis*, as well as to all other mammalian species infecting humans. This led to the adaptation and continuous propagation of the *C*. *hominis* strain TU502 for over a decade and half. It was originally obtained from a child with diarrhea in sub-Saharan Africa [25]. The genome of TU502 was sequenced twice [25, 26] and the strain was tested in human volunteer challenge [27], displaying clinical characteristics distinct from *C*. *parvum*.

Given that the GB piglet is susceptible to all *Cryptosporidium* species infecting humans—including *C*. *hominis—*which causes acute diarrhea that mimics the disease observed in children, we believe GB piglet model is ideal for preclinical efficacy and safety drug testing against *C*. *hominis* as well as other species. Although used extensively in humans, NTZ and AZR have never been evaluated in an optimized and reproducible model of acute diarrhea against cryptosporidiosis. Here, we report for the first time, the characterization and optimization of the GB piglet model infected with *C*. *hominis*, and treated with single and combinations of NTZ and AZR at doses suitable for human use. Following infection with *C*. *hominis*, the diarrheal disease, and responses of GB piglets to NTZ, AZR, and drug combinations recapitulates the observations in humans, in as much as therapies reduce clinical symptoms but do not completely ameliorate oocyst shedding. These findings validate the GB piglet preclinical model.

## Materials and methods

*C*. *hominis* strain TU502, originated from a Ugandan diarrheic child, has been maintained for over a decade through numerous passages (every 8 to 10 weeks) in GB piglets [28]. TU502 oocysts were purified from gut contents and feces and prepared for the challenge experiments, as described previously [27]). Oocysts with an excystation rate of over 60% were used for challenge.

Forty GB piglets, derived from seven litters by Cesarean section, were maintained inside sterile isolators in accordance with the protocol G2015-29 approved by the Tufts University Institutional Animal Care and Use Committee. Piglets were fed 3 times daily with a total of 500–700 ml/day of human infant milk replacer (Similac). Piglets were monitored a minimum of 3 times each day. Healthy piglets, selected 2 days after birth, were assigned to six experimental groups.

Piglets would be euthanized if they 1) exhibit signs of severe dehydration (>8%; determined by extent of skin elasticity); 2) exhibit signs of wasting based on evidence of inadequate milk intake (<75 mls total volume via handfeeding during a 24 hour period); 3) are unresponsive to handling; 4) become moribund as evidenced by lack of responsiveness to handling, hypothermia, and severe depression; or 5) if piglets are observed to be lethargic or unable to move in response to tactile stimulation. No piglet was euthanized earlier with the serious symptoms of illness. However, some piglets were found dead in the duration of study (Two piglets were found dead on day 6 and day 8 post NTZ treatment, respectively; Two piglets were found dead on day 3 and day 9 post AZR and NTZ treatment, respectively. The data from the piglets found dead under day 6 were excluded for analysis). At the end of study, piglets were euthanized by intracardiac administration of phenytoin/pentobarbital (1 ml/4.5 kg) following intramuscular administration of ketamine (100 mg/kg) and xylazine (5 mg/kg).

TU502 1-5x10^6^ oocysts purified from gut contents and feces were administered orally 2 days after birth. The infected piglets were treated with drugs at onset of diarrhea 3 days after the *C*. *hominis* challenge. AZR (Teva pharmaceuticals, North Wales, PA) and NTZ (Alinia®, Lupin pharmaceuticals, Baltimore, MD) were used at the recommended pediatric doses: AZR at 50 mg in 2.5 ml suspension once a day for 10 days; NTZ at 100 mg in 5 ml suspension was given twice a day for 10 days. For the combined treatment group, AZR at 50 mg in 2.5 ml suspension and NTZ at 100 mg in 5 ml suspension, were administered once a day and twice a day, respectively, for 10 days.

Clinical signs of illness were monitored twice daily and symptoms of diarrhea were scored from 0 to 4 (0 –no diarrhea; 1 –brown or grey colored, soft stool, mild diarrhea; 2 –brown to yellow colored, mucoid, small volumetric stool, mild to moderate diarrhea; 3 –yellow colored, mucoid to watery, medium volumetric stool, moderate diarrhea; 4 –yellow to white colored, watery, high volumetric stool, severe diarrhea). Two lab personnel monitored and scored individually and averaged. Body weight was measured daily.

Rectal swabs were collected daily while sera were collected on day 1, 8, and 10 once drug treatment commenced. Daily rectal swabs were placed in 500 μl sterile H_2_O, incubated at room temperature for 10 min; agitated eluates were centrifuged at 13,000 x g for 10 min to separate the oocysts in the pellet from the residual drugs and their metabolites in supernatant. 300 μl of supernatant was transferred to a new tube and stored at -80°C to be used for measuring drug concentration and/or their metabolites. The pellet was resuspended, washed and pelleted and resuspended in the remaining 50 μl of which 10 μl was mounted on welled glass slides (diameter 7 mm); and stained by the modified Kinyoun acid fast method [29]. The number of oocysts were counted in 30 fields under 1,000x magnification. The number of oocysts were counted independently by two lab individuals and averaged.

Serum and rectal swabs were selected from two representative animals per experimental group to measure the residual drugs and their metabolites. The concentrations of AZR and NTZ, and NTZ active metabolite, tizoxanide (TIZ) and TIZ-glucuronide, in samples were measured by mass spectrophotometry. In vitro drug potency for NTZ and AZR was perform as previously described for *C*. *parvum* [30].

Piglets were euthanized after 10 days of treatment, or earlier if they showed serious symptoms of illness. Blood samples were collected and sent to Tufts Clinical Pathology Laboratory for hematology and serum biochemical data analysis. At necropsy, tissue for histology were collected from the small and large intestines, stomach, liver, kidney, lung, spleen, brain, and gonads, processed and examined by a board certified veterinary pathologist. The extent of *C*. *hominis* colonization was scored in small and large intestines as described previously: 0, no parasite forms detected; 1, 1–20% epithelial surface infected; 2, 21–40% surface infected; 3, 41–60% surface infected; 4, 61–80% surface infected; 5, 81–100% surface infected [31]. Similar scores were developed for epithelial dysplasia, reduction in goblet cells, and lymphocytic inflammation, using minimal = 0, mild = 5, moderate = 10 or marked = 15, or severe = 20. The overall pathology score was a sum of scores from the ileum and large intestine for each individual piglet. All scores were acquired from three sites of each intestinal section and averaged.

The statistical comparison of the differences among experimental groups was conducted using the statistical tools in GraphPad Prism 7.01 software (GraphPad Software, San Diego, CA, USA).

## Results

### The model

**Fig 1:**
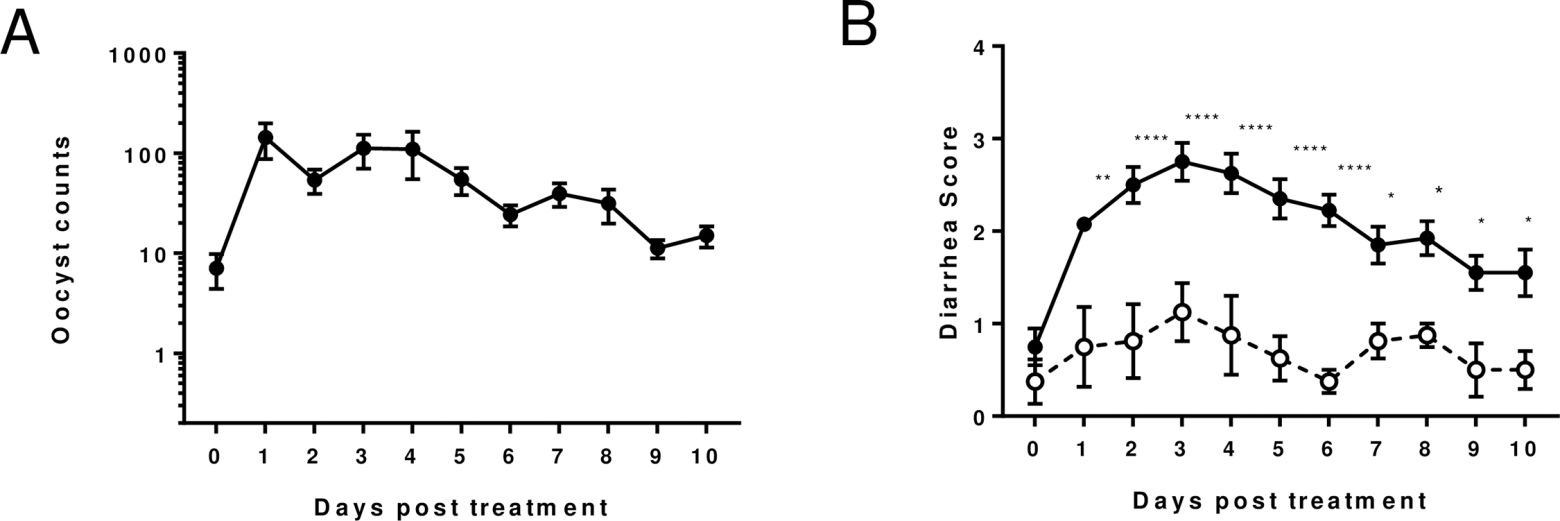
The oocyst excretion pattern, intensity of diarrhea and body weight gains of the GB piglets orally challenged with TU502 oocysts. (A) Daily oocyst shedding (means ± SEM) in feces. The rectal swabs were processed and counted in 30 microscopic fields under 1,000x magnificent. (B) Daily diarrhea scores (means ± SEM) observed in piglets. Symptoms were monitored and scored daily by 2 individuals; diarrhea was scored from 0 to 4 (0 –no diarrhea; 1 –brown, grey, soft stool, mild diarrhea; 2 –brown yellow, mucoid, mild to moderate diarrhea; 3 –yellow, mucoid to watery, moderate diarrhea; 4 –white to yellow, watery, severe diarrhea). Two-way ANOVA with Sidak's multiple comparisons test was performed using GraphPad Prism 7.01. *p<0.05, **p<0.01, ****p<0.0001. TU502-infected group (n = 10, closed circle ●); Uninfected control (n = 4, open circle ○). Loose feces in uninfected animals, attributed to the milk diet, are often observed.

GB piglets challenged with *C*. *hominis* TU502 excreted oocysts in feces within 3 days after challenge and continued to do so, regardless of treatment, until they were euthanized at the end of the 10-day treatment experiment. The oocyst excretion pattern, intensity of diarrhea and body weight gains of the infected group, as compared with the uninfected control group, are reflected in Fig 1. Excretion peaked over the first 4 days after challenge, declining gradually thereafter (Fig 1A). Infected piglets developed mild to moderate diarrhea lasting 7 to 10 days. Moderate diarrhea tended to occur 3 to 5 days after onset (Fig 1B).

### Impact of treatment

**Fig 2:**
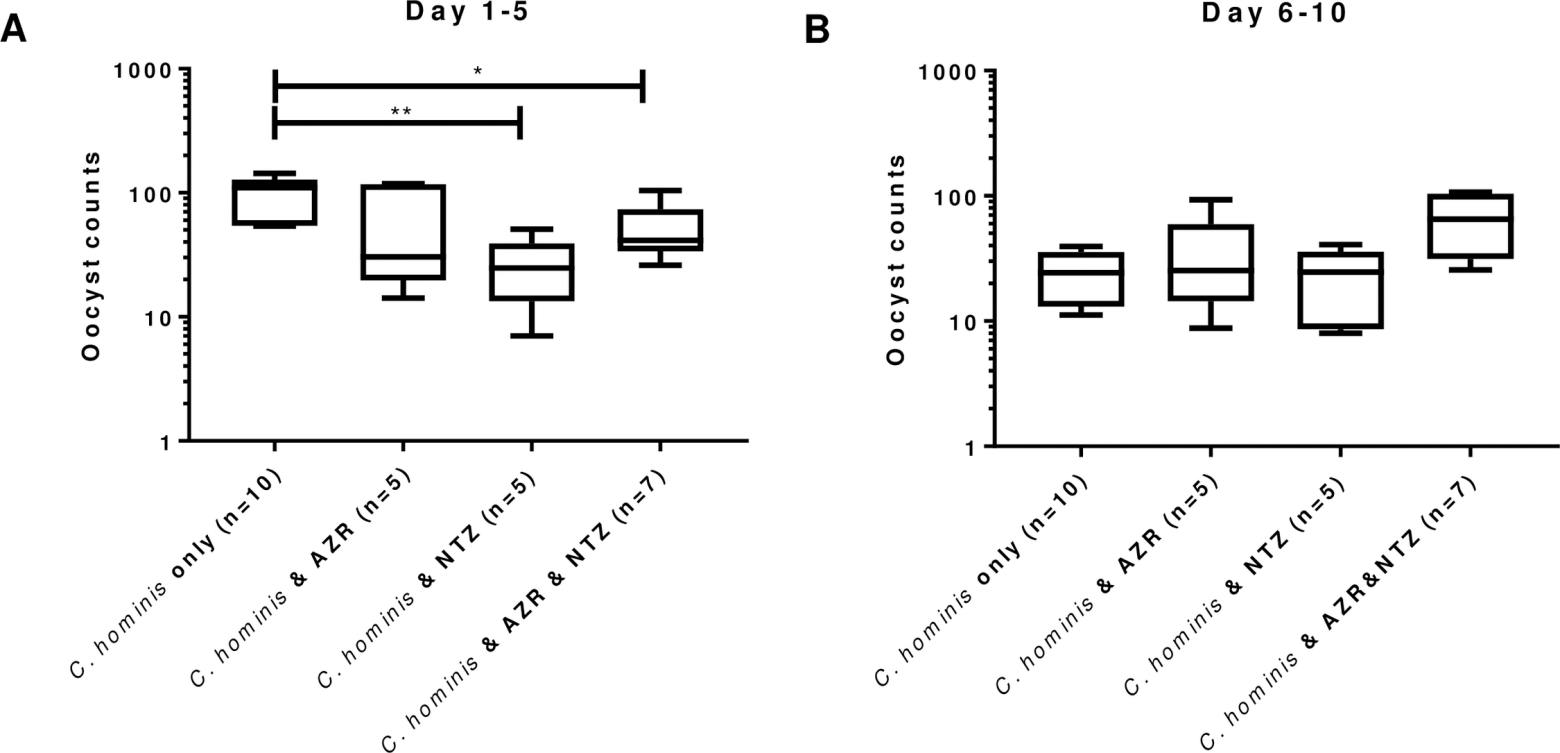
Accumulated counts of oocyst excretion in feces of TU502-challenged piglets treated with either, AZR, NTZ or AZR+NTZ, as compared with an untreated infected group. There was a notable contrasting in the level of oocyst excretion between days 1–5 (A) and day 6–10 (B) of treatment, as shown with box-and-whiskers plot (min to max). Mann-Whitney test was conducted using GraphPad Prism 7.01. *p<0.05, **p<0.01.

NTZ treatment reduced the oocyst count of *C*. *hominis*-infected piglets, compared to infected control. However, the difference of the daily oocyst shedding was not statistically significant (results not shown). The accumulated counts of oocyst shedding (Fig 2) indicated that shedding was significantly reduced in piglets treated with either NTZ or with AZR+NTZ on day 1–5 post treatment (A), but not day 6–10 (B) of treatment. In fact the accumulated excretion in piglets treated with AZR+NTZ had higher shedding rate of oocysts on days 6–10 compared to the untreated group in Fig 2B (p = 0.0556), AZR treatment had no significant impact on the level of oocyst excretion over the 10 day treatment.

**Fig 3:**
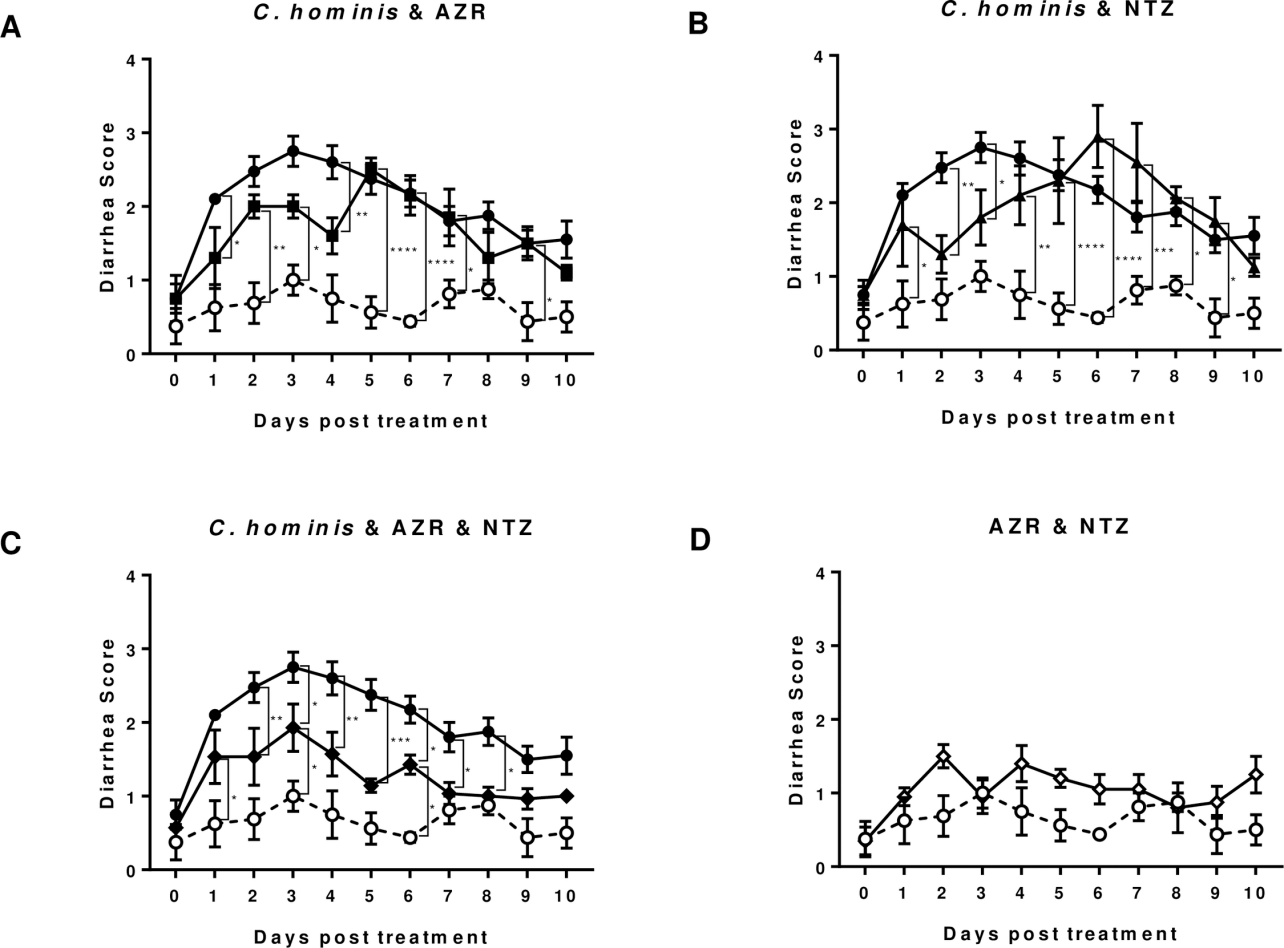
**Daily diarrhea scores observed in piglets treated with either AZR (A), NTZ (B) or AZR+NTZ (C), compared with uninfected AZR+NTZ-treated and untreated groups (D).** Two-way ANOVA with Tukey's multiple comparisons test was conducted using GraphPad Prism 7.01. *p<0.05, **p<0.01, ***p<0.001, ****p<0.0001. TU502-infected group (n = 10, closed circle ●) was compared with AZR-treated group (n = 5, closed squire ■) in A; with NTZ-treated group (n = 5, closed triangle ▲) in B; and with AZR+NTZ-treated group (n = 7, closed rhombus ◆) in C; Uninfected group treated with AZR+NTZ (n = 5, open rhombus ◇) and untreated control group (n = 4, open circle ○) are presented in D.

The daily diarrhea scores (Fig 3) showed that treatment with AZR (Fig 3A) or NTZ (Fig 3B) reduced the diarrhea for the first few days, but the effects were transient and efficacy was lost after 5 days and reverted to the level of the untreated control animals. In contrast, the combined AZR+NTZ treatment significantly reduced diarrhea in parasite-infected piglets (Fig 3C). This correlates well with the accumulated oocyst counts seen in Fig 2. Drug-related mild diarrhea was observed in uninfected piglets treated with AZR+NTZ (Fig 3D).

**Fig 4:**
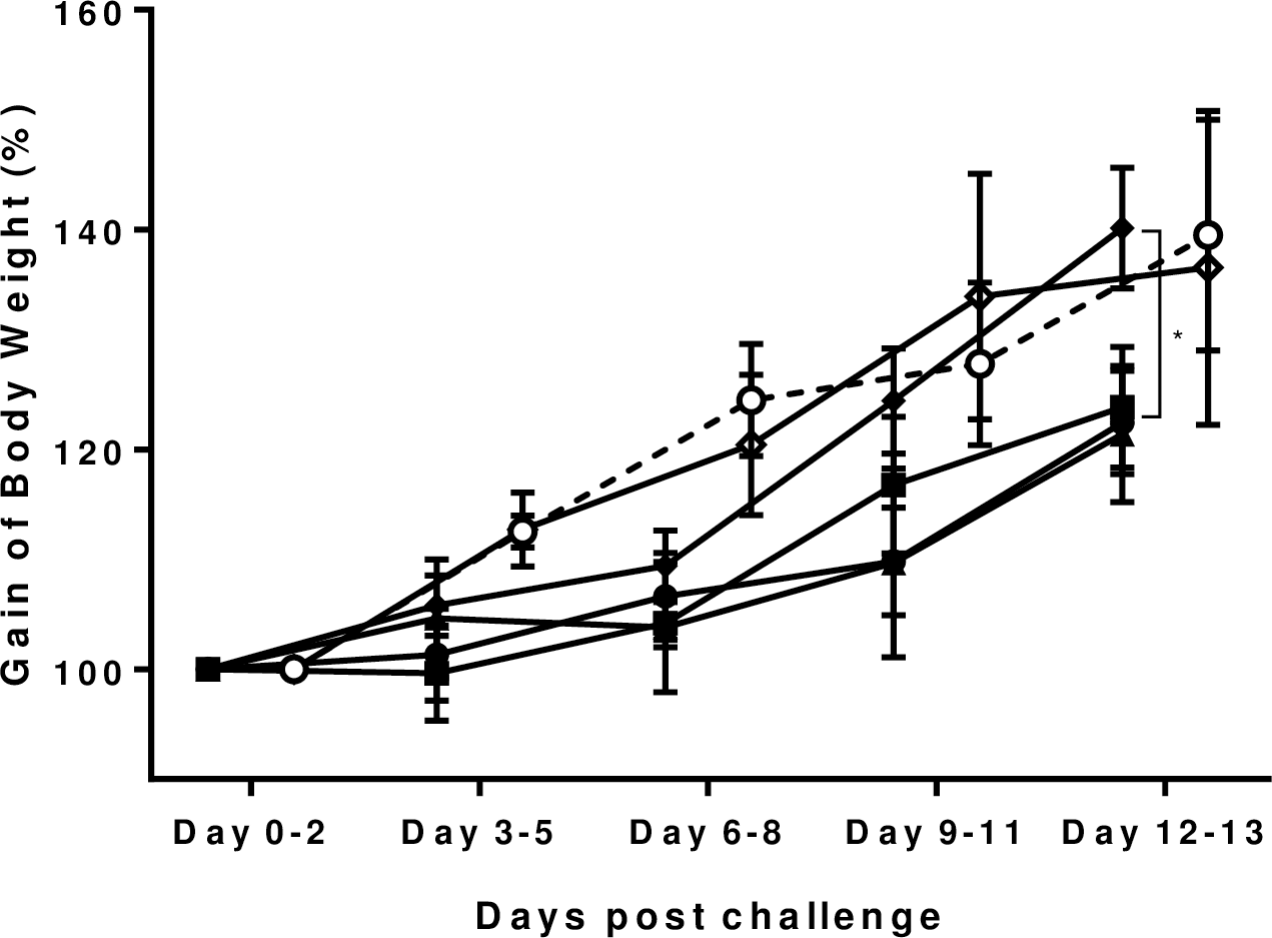
Gaining of body weight after drug treatment for *C*. *hominis*-infected piglets. Gnotobiotic piglets were inoculated orally with C. hominis oocysts two days after birth and treated with AZR, NTZ, or combined AZR and NTZ three days post challenge. Body weight was measured daily. Two-way ANOVA with Tukey’s multiple comparisons test was conducted using Graphpad Prism 7.01. * p<0.05. *C*. *hominis* TU502-infected group (n = 7, closed circle ●); TU502-infected, AZR-treated group (n = 3, closed squire ■); TU502-infected, NTZ-treated group (n = 3, closed triangle ▲). The partial measurement from a piglet, which was dead on day 8 post NTZ treatment were included; TU502-infected, AZR+NTZ-treated group (n = 7, closed rhombus ◆); Uninfected, AZR+NTZ treated group (n = 3, open rhombus ◇); and untreated control group (n = 2, open circle ○). The *C*. *hominis* TU502-infected group and untreated control group already shown in Fig 1 were added for comparison. The measurement of body weight from experiment I, II, and III was excluded due to the inaccuracy of scale.

Body weight gain, measured daily, showed that infected piglets tended to have lower body weight gain compared to uninfected control animals and a statistically significant improvement was observed in the infected piglets treated with AZR+NTZ vs. the infected control (p<0.05) (Fig 4).

### Microscopic lesions

**Fig 5:**
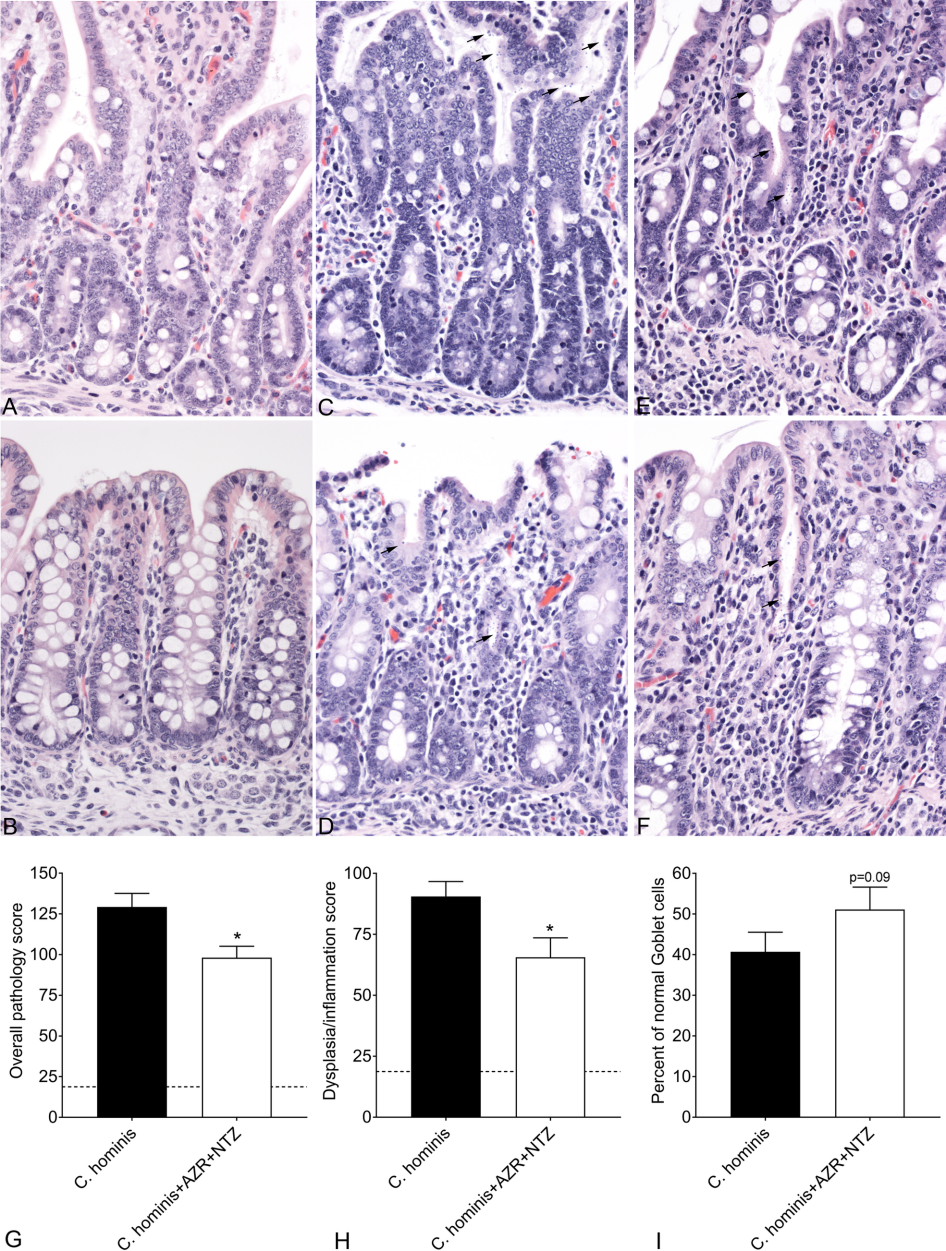
Microscopic lesions of *C*. *hominis* infection. Photomicrographs are from ileum and large intestine, stained with hematoxylin and eosin, magnified 400 times normal. Normal small and large intestine are shown in A and B, respectively. Small and large intestine from *C*. *hominis* are shown in C and D, respectively with moderate epithelial dysplasia, moderate loss of Goblet cells, moderate lymphocytic inflammation, and *C*. *hominis* organisms (black arrows). Small and large intestine from *C*. *hominis* treated with ARZ+NTZ are shown in E and F, respectively with mild epithelial dysplasia, mild loss of Goblet cells, and *C*. *hominis* organisms (black arrows). The overall pathology score is shown in G, with component scores for dysplasia/inflammation and Goblet cells shown in H and I, analyzed by the Mann-Whitney *t-*test with p<0.05. Dashed lines indicate the average score for uninfected piglets.

Piglets were euthanized 13 days after the oral challenge. As expected, *C*. *hominis* infection was mostly observed in distal small and large intestines, and there was a modest reduction in the epithelial colonization score attributable to AZR, NTZ, and AZR+NTZ (not shown), also consistent with oocyst excretion in the feces. As the AZR+NTZ treatment reduced the diarrhea score and oocyst shedding, microscopic examination focused on the combination treatment (Fig 5). Photomicrographs of small intestine and large intestine from a non-infected pig are shown for comparison (Fig 5A and 5B). Examples of small intestine and large intestinal lesions due to *C*. *hominis* (Fig 5C and 5D) are shown. Small and large intestinal lesions from a piglet infected with *C*. *hominis* and then treated with AZR+NTZ are shown in Fig 5E and 5F, respectively. The overall pathology score (Fig 5G) was significantly reduced by AZR+NTZ treatment as compared to piglets challenged with *C*. *hominis* alone. This score is an aggregate reflecting dysplasia/inflammation (Fig 5H) and Goblet cells (Fig 5I), and parasite infection (not shown). These results indicate that *C*. *hominis* infection of GB piglets induces similar lesions as observed in humans with *C*. *hominis* and that treatment with ARZ+NTZ improves but does not completely ameliorate epithelial damage, mucosal lymphocytic inflammation, and parasite colonization.

Pharmacokinetic data are summarized in Table 1, measured in sera and feces of the 4 experimental groups. While it was possible to directly measure the concentrations of AZR and NTZ; NTZ samples were also monitored for the active metabolites TIZ and TIZ-glucuronide. AZR in sera was 1.9 μg/ml (median 2.0 μg/ml) and 0.4 μg/ml (median 0.3 μg/ml) in rectal swabs. NTZ levels were under the quantitation limit in sera and rectal swabs, as it instantly was converted to its active metabolites TIZ and TIZ-glucuronide after oral administration. TIZ levels were 8.0 μg/ml (median 1.4 μg/ml) in sera and 6.5 μg/ml (median 4.0 μg/ml) in fecal swabs, while TIZ-glucuronide’s levels were 7.8 μg/ml (median 5.3 μg/ml) in sera and 31.0 μg/ml (median 33.8 μg/ml) in rectal swabs. TIZ-glucuronide concentration in rectal swabs was 4- to 6-fold higher than TIZ concentration. While AZR concentration in sera was higher in infected piglets treated with AZR alone than in infected or uninfected piglets treated with AZR+NTZ (2.3 μg/ml vs 1.5 μg/ml or 1.8 μg/ml), the drug concentration in rectal swabs was significantly lower in piglets treated with AZR alone vs. parasite-infected or uninfected piglets with AZR+NTZ (0.2 μg/ml vs 0.4 μg/ml or 0.5 μg/ml; Mann Whitney test p = 0.0007 and p = 0.0052, respectively) (Table 1). There was no significant difference in the concentration of AZR observed in sera and rectal swabs between infected and uninfected piglets treated with AZR+NTZ.

**Table 1:** Concentration of AZR and NTZ, plus metabolites TIZ and TIZ-glucuronide in sera and rectal swabs.

	Mean ± SEM (median), mg/ml		
	AZR	TIZ	TIZ-glucuronide
Serum			
*C*. *hominis* & AZR	2.3 ± 0.16 (2.5)		
*C*. *hominis* & NTZ		15.6 ± 8.63 (4.1)	9.9 ± 4.00 (4.7)
*C*. *hominis* & AZR+NTZ	1.5 ± 0.35 (1.4)	6.7 ± 6.35 (0.3)	5.0 ± 1.72 (4.3)
Control AZR+NTZ	1.8 ± 0.23 (1.8)	1.7 ± 0.46 (1.4)	8.6 ± 2.13 (6.1)
Rectal swab eluate			
*C*. *hominis* & AZR	**0.2 ± 0.04 (0.2)**		
*C*. *hominis* & NTZ		4.6 ± 0.63 (4.4)	35.8 ± 3.67 (40.7)
*C*. *hominis* & AZR+NTZ	0.4 ± 0.04 (0.4)	4.9 ± 0.94 (4.2)	24.0 ± 3.52 (27.4)
Control AZR+NTZ	0.5 ± 0.09 (0.3)	10.1 ± 3.02 (6.1)	33.1 ± 4.98 (50.0)

AZR concentration in rectal swabs was significantly lower in parasite-infected piglets treated with AZR alone (bold) vs. parasite-infected or uninfected piglets with AZR+NTZ (underlined) (Mann Whitney test p = 0.0007 and p = 0.0052, respectively)

TIZ concentration in sera was higher in infected piglets treated with NTZ alone [15.6 μg/ml (median 4.1 μg/ml)] vs. uninfected piglets given AZR+NTZ [1.7 μg/ml (median 1.4 μg/ml)] (Table 1). In contrast, TIZ concentration in rectal swabs was higher in uninfected piglets with AZR+NTZ [10.1 μg/ml (median 6.1 μg/ml)] than parasite-infected piglets with NTZ alone [4.6 μg/ml (median 4.4 μg/ml)] (Table 1). TIZ-glucuronide concentration was similar among the groups.

The growth of *C*. *hominis* TU502 in MDBK cell culture was inhibited at a concentration of 2 μg/ml of NTZ and 200 μg/ml of AZR, respectively.

Serum biochemical and hematological data from blood samples at euthanasia of the piglets, involving some 35 parameters were analyzed (data not included), and compared to the reference data from domestic pigs or specific pathogen free pigs [32–36]. Because the GB piglets used in this study are quite a distinct group from the reference populations, being immunologically immature, at a very young age, raised differently and fed an artificial diet, we have opted instead to focus on differences within the 6 experimental groups (Table 2).

**Table 2:** Serum biochemical data and hematology of gnotobiotic piglets used in this study.

Measure	Units	*C*. *hominis-*infected groups				Control	
		control (n = 10)	NTZ (n = 4)	AZR (n = 5)	AZR+NTZ (n = 7)	none (n = 4)	AZR+NTZ (n = 4)
Chloride	mEq/L	92 ± 0.9	92 ± 3.0	95 ± 0.7	90 ± 1.4	98 ± 0.9	93 ± 4.2
Anion Gap	mmol/L	19 ± 0.9	**15 ± 1.9**	**15 ± 0.6**	**16 ± 0.9**	21 ± 0.6	**15 ± 1.2**
Phosphorus	mg/dL	8.6 ± 0.28	**6.9 ± 0.24**	7.3 ± 0.47	**7.2 ± 0.25**	8.6 ± 0.53	7.5 ± 0.29
Creatinine	mg/dL	**0.4 ± 0.03**	0.5 ± 0.06	0.4 ± 0.02	**0.4 ± 0.03**	0.5 ± 0.05	0.6 ± 0.07
Bilirubin	mg/dL	0.4 ± 0.06	0.3 ± 0.08	0.4 ± 0.04	**0.1 ± 0.03**	0.5 ± 0.03	0.2 ± 0.06
Cholesterol	mg/dL	97 ± 5.6	**138 ± 7.9**	108 ± 6.9	114 ± 5.5	94 ± 3.4	112 ± 9.1
ALT	U/L	59 ± 3.4	52 ± 8.9	54 ± 1.7	**43 ± 2.6**	52 ± 2.5	45 ± 3.5
Amylase	U/L	3036 ± 197	2436 ± 396	3095 ± 455	**2004 ± 195**	3755 ± 212	**1959 ± 407**
Total protein	g/dL	2.4 ± 0.14	2.4 ± 0.09	2.2 ± 0.12	2.3 ± 0.13	1.9 ± 0.03	2.2 ± 0.14
Albumin	g/dL	1.4 ± 0.07	1.5 ± 0.13	1.4 ± 0.07	1.3 ± 0.06	1.0 ± 0.04	1.2 ± 0.1
Hematocrit	%	25.0 ± 0.97	25.0 ± 1.35	23.2 ± 0.97	25.4 ± 1.07	29.0 ± 0.41	**20.3 ± 2.96**

The data represent mean ± SEM. Mann Whitney test was conducted between *C*. *hominis*-infected piglets and non-treated piglets using Graphpad Prism 7.01. In *C*. *hominis*-infected piglets, the chloride level was significantly lower than those of non-infected piglets (p = 0.002). Total protein and albumin were significantly higher than those of non-infected piglets (p = 0.038 and p = 0.005, respectively). Hematocrit was significantly lower than those of non-infected piglets (p = 0.027).

Tukey's multiple comparisons test among the treatment groups was conducted using GraphPad Prism 7.01. Anion gap was significantly lower in AZR and/or NTZ-treated piglets (bold) than untreated control piglets (underlined). Phosphorous was significantly lower in *C*. *hominis*-infected NTZ and NTZ+AZR-treated piglets (bold) than *C*. *hominis*-infected control (underlined). Creatinine was significantly lower in *C*. *hominis*-infected control and *C*. *hominis*-infected AZR+NTZ treated groups (bold) compared with AZR+NTZ-treated control piglets (underlined). Bilirubin was significantly lower in *C*. *hominis*-infected, AZR+NTZ-treated piglets (bold) than *C*. *hominis*-infected control and untreated control groups (underlined). Cholesterol was significantly higher in infected, NTZ-treated piglets (bold) than untreated infected control piglets and untreated control piglets (underlined). ALT was significantly lower in *C*. *hominis*-infected, AZR+NTZ-treated piglets (bold) than the infected control group (underlined). Amylase was significantly lower in AZR+NTZ treated piglets (bold) than untreated control piglets (underlined). Hematocrit was significantly lower in AZR+NTZ-treated control piglets (bold) than untreated control piglets (underlined). ALT: alanine amino-transferase

*C*. *hominis* infection altered some blood biochemical and hematological parameters although most parameters were not affected (data not shown). In *C*. *hominis*-infected piglets, chloride 92 ± 0.9 mEq/L was significantly lower than 98 ± 0.9 mEq/L of non-infected piglets and reference range 93–108 mEq/L. Creatine 0.4 ± 0.03 mg/dL was lower than reference range 0.5–2.7 mg/dL, however there was no significant difference between *C*. *hominis*-infected piglets and uninfected piglets. Triglycerides 56 ± 0.03 mg/dL were lower than 91 ± 20.1 mg/dL of non-infected piglets. Total protein and albumin at 2.4 ± 0.14 g/dL and 1.4 ± 0.07 g/dL, respectively, in the infected group were significantly higher than those of uninfected piglets, reflecting perhaps a mild state of dehydration. The hematocrit at 25.0 ± 0.97%, was significantly lower than 29 ± 0.41% compared to uninfected piglets, however both were within the reference range. The main change attributable to infection was the reduction in chloride, which likely reflects electrolyte loss through the gastrointestinal tract as a result of *C*. *hominis-*induced diarrhea.

AZR and/or NTZ also altered some blood biochemical and hematological parameters (Table 2). In AZR and/or NTZ-treated piglets, anion gap, and phosphorous ion were lower and cholesterol was higher than those in non-treated piglets. In NTZ-treated piglets, bilirubin and amylase were lower than those in non-treated or sole AZR-treated piglets. Bilirubin and amylase in both AZR and NTZ treated piglets were significantly lower than parasite-infected & untreated piglets. Alanine amino-transferase (ALT) in both AZR and NTZ-treated piglets was lower than those in challenged & untreated piglets. Anion gap, phosphorous, bilirubin, and ALT in all groups were in the reference range. There was no reference for amylase.

Mortalities (euthanized due to severe illness or found dead) were observed among piglets treated with, either NTZ (2 of 6 challenged), AZR+NTZ (1of 8 challenged; 1 of 5 uninfected), and none among AZR-treated animals. Higher mortalities occurred among the groups receiving double the dose of either NTZ (2 of 4) or AZR+NTZ: (4 of 4). There was again, no mortalities among piglets given double the AZR dose, indicating the lack of AZR toxicity even at a higher dose. This is in contrast to the higher level of toxicity of NTZ for piglets at doses higher than the recommended pediatric dose.

Tissue toxicity of NTZ alone and or combined with AZR, investigated microscopically, showed variable, moderate to marked, hepatocellular cytoplasmic vacuolation in the liver; an indicator of metabolic and hepatocyte function. There were no significant differences among the 4 experimental groups, regardless of treatment. The exception was the group which had received double the NTZ dose, suggesting that NTZ may alter hepatocyte function directly or indirectly through an effect on metabolism elsewhere in the body. No significant microscopic lesions were observed in any of the other extraintestinal tissues examined reflecting lack of toxicity associated with either drug or the combination thereof.

## Discussion

These studies were undertaken to characterize and optimize key parameters of the GB piglet model of *C*. *hominis*. They include acute diarrheal illness, oocyst excretion in the feces, body weight gain (Figs 1 and 4), nature and extent of mucosal lesions and parasite infection, pharmacokinetics and drug toxicity, blood chemistry and hematology (Tables 1 and 2). These results highlight the range of assays that can readily be performed in this animal model which would assist in the overall evaluation and safety of therapeutic agents. The GB piglet model was optimized for the purpose of evaluating future prophylactic and therapeutic agents against cryptosporidiosis for potential use in children, using two well documented FDA-approved drugs known to have some therapeutic benefits against the infection. Beside humans, *C*. *hominis* experimentally infects only piglets, which unlike the mammalian species *C*. *parvum* which infects all mammalian species including humans and rodent models. As commonly seen in children, *C*. *hominis* induces acute self-limited diarrheal illness lasting 7–10 days in piglets. The piglet model, in fact, is susceptible to all the species that have commonly been reported to infect humans, namely *C*. *parvum* (bovine), *C*. *parvum* (anthroponotic), and *C*. *meleagridis*. Although no animal model perfectly mimics the human condition, the piglet has emerged as a premier experimental animal model suitable for investigations of several enteric infections in the pediatric population because of size, age, anatomic, physiologic and digestive similarities, and dietary requirements; GB piglets are fed the infant formula “Similac” [28, 31, 37–40]. Additional advantages of the GB piglet model, apart from its susceptibility to infections, while labor intensive, includes a reproducible outcome, in a highly controlled environment, in which litters of ~10–12 derived by cesarean are born agammaglobulinemic, free of interfering maternal antibodies, and can be divided into several age- and size-matched experimental groups.

*C*. *hominis* contributes to >75% of *Cryptosporidium* cases in African and Asian infants over the first 18 months of life, subsiding thereafter [1, 2]. The observed symptoms in piglets while picked up 5–7 days after the onset of diarrhea, the infection often continues for 2–3 weeks (data not shown), as evident by continued oocyst excretion and presence of parasite forms in the gut mucosa. The GB piglet model is ideal for evaluating drugs against acute cryptosporidiosis as was shown in this study using either a single drug or a combination of two drugs. Consistent with observations in children and adults with cryptosporidiosis treated with NTZ, the outcome in the piglet model was beneficial in reducing the severity of diarrhea and oocyst excretion in the feces only initially but failed to clear the infection. Our study showed that either increasing the course of treatment to 10 days or doubling the NTZ dose did not improve the outcome, and was poorly tolerated. A combined AZR+NTZ therapy had a moderate initial impact on the clinical and parasitological outcome as predicted by clinicians and reported to have a synergistic effect in vitro experiments [24].

AZR treatment over 10 days had no effect on the level of oocyst excretion, in contrast to NTZ treatment which had significant reduction of oocysts excretion in the early phase of treatment. The AZR+NTZ drug treatment was associated with resolution of diarrhea but failed to eliminate oocyst excretion or significantly impact the degree of mucosal injury observed at necropsy. In reports on the use of NTZ in people, NTZ treatment resulted in a significant oocyst clearance compared with placebo, however it was ineffective in reducing duration and frequency of diarrhea and mortality in adults and children [41]. AZR treatment for cryptosporidial diarrhea in AIDS patients was associated with good clinical improvement but was ineffective in eradicating the infection [19].

The growth of *C*. *hominis* TU502 in MDBK cell culture was inhibited at a concentration of 2 μg/ml of NTZ and 200 μg/ml of AZR, respectively. Although the in vitro potency of NTZ metabolites, TIZ and TIZ-glucuronide were not evaluated for *C*. *hominis* TU502, the previous report indicated that 1.2 μg/ml of NTZ and 2.2 μg/ml of TIZ-glucuronide resulted in a 50% inhibition of *C*. *parvum* in HCT-8 cells [42].

Consequently, the concentration of AZR, NTZ, and its metabolites in rectal swab eluate indicate that the concentration of TIZ-glucuronide (24 mg/ml) and AZR (0.4 mg/ml) in the gut would be enough to inhibit *C*. *hominis*. However, we observed the limited reduction of oocyst excretion in piglets treated with AZR or NTZ alone, or the combination of the two. These observations are similar with a previous study, which showed that NTZ was partially effective at reducing parasite burden in the gnotobiotic piglet diarrhea model when given orally for 11 days at 250 mg/kg/day [30], suggesting that the potency seen in the in vitro studies do not reflect observations made in the piglet model.

Side-effects associated with NTZ treatment in humans include decreased liver function, discolored urine, and hives [9]. Mild gastrointestinal side effects were also reported after oral dosing of NTZ [8]. Treatment of piglets with AZR+NTZ led to drug induced loose stool/mild diarrhea. The higher dose of NTZ may have also induced a drug-related diarrhea in piglets that might have influenced its therapeutic efficacy [30].

There were some sudden deaths associated with NTZ treatment increasing with doubling the daily dose to > 200 mg/twice daily, and with doubling the AZR+NTZ dose. A similar acute toxicity of NTZ was observed in goats [43], when NTZ treatment was given at a daily dose of 100 mg/kg for 7 days or 200 mg/kg for 8 days in 2- to 4-day-old goat kids, 5 out of 16 kids (31%) and 7 out of 15 kids (47%) respectively died, with respiratory distress 24 to 48 h after the first NTZ administration at both 100 and 200 mg/kg dosing [43]. Mild respiratory distress was also observed in piglets treated with NTZ, however no histological abnormalities were detected in the lung at necropsy. In a previous study reported by our group evaluating the impact of NTZ treatment in GB piglets given 250mg/kg/day, after challenge with *C*. *parvum*, two of 5 piglets were euthanized on day 5 and 6 after the onset of treatment [30], indicating that high NTZ dose can be toxic to newborn piglets. Its use therefore in infants may require additional scrutiny. In contrast, no safety issues were identified with higher doses of AZR in our study.

In summary, we show that the GB piglet is a useful model to evaluate the safety and efficacy of new therapeutic agents for infant and children against *C*. *hominis* as well as against all other species associated with cryptosporidiosis in people. The versatility of the piglet model, beginning with its susceptibility, its infancy and size matching the pediatric population, and the ability to measure the extent and duration of diarrhea and oocysts excretion, body weight gain, degree of mucosal injury at necropsy, pharmacokinetics, tissue toxicity, blood chemistry and hematology, makes this model an excellent tool to evaluate therapeutic agents. Although NTZ and AZR are extensively used in humans, their therapeutic efficacy has never been evaluated systematically and in combination in an optimized reproducible animal model of acute cryptosporidiosis. Some clinical benefit was observed in the early phase of treatment with NTZ, less with AZR, in piglets challenged with *C*. *hominis*. A significant symptomatic improvement however was observed in piglets treated with the AZR+NTZ combination, with an initial reduced oocyst excretion, reflecting a failure to eliminate the infection. The study in particular demonstrates that increasing the dose or the duration of treatment are of little therapeutic benefit.

## Supporting information

S1 Fig Comparison between oocyst counts and quantification of oocyst DNA using quantitative real time PCR. The oocyst counting method was modified to monitor daily oocyst shedding in this study. To verify the oocyst count method, the oocyst count results from representative samples were compared with the quantitative DNA measurement using real time PCR. *C*. *hominis* TU502-infected group (n = 2, closed circle ●); TU502-infected, AZR-treated group (n = 2, closed squire ■); TU502-infected, NTZ-treated group (n = 2, closed triangle ▲); TU502-infected, AZR+NTZ-treated group (n = 2, closed rhombus ◆).
(DOCX)

S1 Table Oocyst excretion in feces of TU502-challenged piglets treated with either AZR, NTZ, or AZR+NTZ, as compared with an untreated infected group. (DOCX)

S2 Table Daily diarrhea scores observed in piglets treated with either AZR, NTZ, or AZR+NTZ, as compared with uninfected AZR+NTZ treated and untreated groups. (DOCX)
