# Supplementary material for: The therapeutic efficacy of azithromycin and nitazoxanide in the acute pig model of Cryptosporidium hominis
Source: PLoS One. 2017 Oct 3;12(10):e0185906. doi: 10.1371/journal.pone.0185906 (PMC5626496; doi:10.1371/journal.pone.0185906)
Supplement: S1 Fig — The oocyst counting method was modified to monitor daily oocyst shedding in this study. To verify the oocyst count method, the oocyst count results from representative samples were compared with the quantitative DNA measurement using real time PCR. C. hominis TU502-infected group (n = 2, closed circle ●); TU502-infected, AZR-treated group (n = 2, closed squire ■); TU502-infected, NTZ-treated group (n = 2, closed triangle ▲); TU502-infected, AZR+NTZ-treated group (n = 2, closed rhombus ◆). (DOCX) [file pone.0185906.s001.docx]

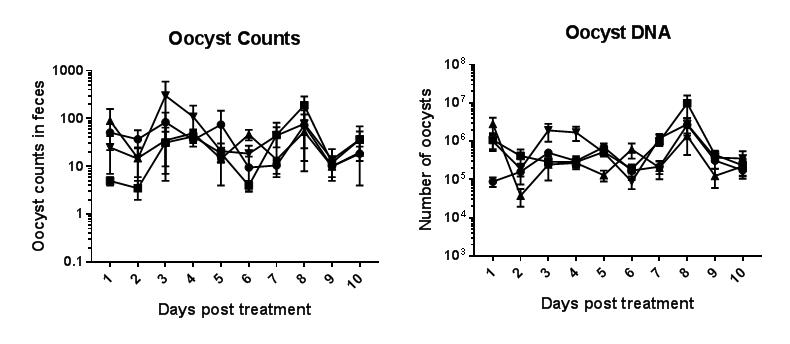


**S1 Fig. Comparison between oocyst counts and quantification of oocyst DNA using quantitative real time PCR.** The oocyst counting method was modified to monitor daily oocyst shedding in this study. To verify the oocyst count method, the oocyst count results from representative samples were compared with the quantitative DNA measurement using real time PCR. *C. hominis* TU502-infected group (n=2, closed circle ●); TU502-infected, AZR-treated group (n=2, closed squire ■); TU502-infected, NTZ-treated group (n=2, closed triangle ▲); TU502-infected, AZR+NTZ-treated group (n=2, closed rhombus ⯁).
