# Supplementary material for: The therapeutic efficacy of azithromycin and nitazoxanide in the acute pig model of Cryptosporidium hominis
Source: PLoS One. 2017 Oct 3;12(10):e0185906. doi: 10.1371/journal.pone.0185906 (PMC5626496; doi:10.1371/journal.pone.0185906)
Supplement: S1 Table — (DOCX) [file pone.0185906.s002.docx]

**S1 Table.** Oocyst excretion in feces of TU502-challenged piglets treated with either AZR, NTZ, or AZR+NTZ, as compared with an untreated infected group.

| Days post treatment | 1 | 2 | 3 | 4 | 5 | 6 | 7 | 8 | 9 | 10 |
| --- | --- | --- | --- | --- | --- | --- | --- | --- | --- | --- |
| *C. hominis* only (n=10) | 549 | 19 | 17 | 12 | 2 | 9 | 27 | 55 | 9 | 24 |
|  | 51 | 17 | 35 | 42 | 4 | 3 | 7 | 122 | 6 | 18 |
|  | 51 | 57 | 133 | 30 | 146 | 16 | 14 | 24 | 14 | 19 |
|  | 93 | 107 | 246 | 10 | 117 | 15 | 34 | 19 | 17 | 11 |
|  | 337 | 53 | 420 | 138 | 69 | 7 | 41 | 1 | 3 | 1 |
|  | 91 | 133 | 131 | 555 | 44 | 41 | 44 | 4 | 5 | 7 |
|  | 8 | 2 | 17 | 19 | 29 | 49 | 121 | 37 | 5 | 7 |
|  | 0 | 6 | 2 | 34 | 20 | 53 | 58 | 2 | 13 | 39 |
|  | 222 | 39 | 76 | 22 | 9 | 32 | 12 | 6 | 13 | 20 |
|  | 33 | 105 | 42 | 235 | 107 | 18 | 37 | 45 | 27 | 4 |
| *C. hominis* & AZR (n=5) | 6 | 2 | 10 | 59 | 15 | 5 | 25 | 290 | 13 | 69 |
|  | 4 | 5 | 53 | 26 | 23 | 3 | 66 | 91 | 11 | 4 |
|  | 75 | 41 | 470 | 32 | 14 | 8 | 6 | 58 | 12 | 41 |
|  | 431 | 90 | 41 | 8 | 14 | 36 | 18 | 20 | 5 | 7 |
|  | 62 | 14 | 18 | 1 | 5 | 48 | 12 | 7 | 3 | 7 |
| *C. hominis* & NTZ (n=5) | 22 | 24 | 65 | 44 | 15 | 58 | 13 | 93 | 10 | 13 |
|  | 67 | 2 | 11 | 7 | 87 | 9 | 3 | NA | NA | NA |
|  | 159 | 6 | 5 | 55 | 14 | 35 | 15 | 13 | 10 | 26 |
|  | 5 | 1 | 9 | 10 | 13 | 5 | 2 | 11 | 6 | 7 |
|  | 1 | 2 | 9 | 8 | 8 | 16 | 7 | 46 | 11 | 78 |
| *C. hominis* & AZR & NTZ (n=7) | 43 | 25 | 593 | 187 | 12 | 27 | 82 | 148 | 23 | 54 |
|  | 7 | 4 | 7 | 31 | 30 | 10 | 6 | 8 | 5 | 19 |
|  | 100 | 16 | 30 | 11 | 92 | 390 | 330 | 144 | 30 | 32 |
|  | 95 | 11 | 7 | 23 | 110 | 211 | 205 | 38 | 43 | 103 |
|  | 2 | 2 | 6 | 29 | 5 | 37 | 21 | 85 | 42 | 41 |
|  | 24 | 2 | 56 | 2 | 30 | 7 | 71 | 21 | 26 | 7 |
|  | 19 | 123 | 31 | 6 | 22 | 21 | 32 | 11 | 10 | 3 |
