# Supplementary material for: The therapeutic efficacy of azithromycin and nitazoxanide in the acute pig model of Cryptosporidium hominis
Source: PLoS One. 2017 Oct 3;12(10):e0185906. doi: 10.1371/journal.pone.0185906 (PMC5626496; doi:10.1371/journal.pone.0185906)
Supplement: S2 Table — (DOCX) [file pone.0185906.s003.docx]

**S2 Table.** Daily diarrhea scores observed in piglets treated with either AZR, NTZ, or AZR+NTZ, as compared with uninfected AZR+NTZ treated and untreated groups.

| Days post treatment | 0 | 1 | 2 | 3 | 4 | 5 | 6 | 7 | 8 | 9 | 10 |
| --- | --- | --- | --- | --- | --- | --- | --- | --- | --- | --- | --- |
| *C. hominis* only (n=10) | 1.50 | 2.00 | 2.75 | 3.75 | 2.00 | 3.50 | 2.25 | 2.00 | 2.00 | 1.50 | 1.00 |
|  | 0.50 | 1.75 | 1.75 | 3.00 | 2.00 | 2.50 | 1.50 | 1.50 | 1.50 | 1.00 | 1.00 |
|  | 0.25 | 2.50 | 3.00 | 3.25 | 3.00 | 3.00 | 3.00 | 3.00 | 3.00 | 2.25 | 2.00 |
|  | 0.00 | 2.00 | 3.00 | 3.00 | 3.25 | 3.00 | 2.00 | 2.00 | 2.50 | 1.25 | 2.00 |
|  | 1.00 | 2.00 | 1.50 | 1.50 | 3.50 | 1.50 | 2.50 | 1.50 | 1.00 | 1.00 | 3.50 |
|  | 0.25 | 2.50 | 3.00 | 3.00 | 3.00 | 2.50 | 3.00 | 1.00 | 2.25 | 2.50 | 1.00 |
|  | 1.00 | 2.00 | 3.00 | 2.50 | 2.00 | 1.50 | 2.50 | 1.00 | 1.50 | 1.00 | 1.00 |
|  | 0.50 | 2.00 | 3.00 | 3.00 | 3.50 | 2.00 | 2.00 | 2.50 | 1.50 | 2.00 | 1.50 |
|  | 0.50 | 2.00 | 2.00 | 2.50 | 2.00 | 2.00 | 1.50 | 2.00 | 2.00 | 1.00 | 1.50 |
|  | 2.00 | 2.25 | 1.75 | 2.00 | 1.75 | 2.25 | 1.50 | 1.50 | 1.50 | 1.50 | 1.00 |
| *C. hominis* & AZR (n=5) | 0.00 | 1.75 | 1.50 | 2.00 | 2.00 | 2.50 | 1.75 | 1.50 | 2.00 | 1.00 | 1.00 |
|  | 1.25 | 2.50 | 2.00 | 2.50 | 2.00 | 2.50 | 2.00 | 1.25 | 0.75 | 1.50 | 1.00 |
|  | 1.50 | 1.25 | 2.50 | 2.00 | 1.00 | 2.00 | 1.50 | 1.00 | 0.25 | 1.00 | 1.00 |
|  | 1.00 | 0.00 | 2.00 | 1.50 | 2.00 | 2.50 | 2.50 | 3.00 | 2.00 | 2.00 | 1.50 |
|  | 0.00 | 1.00 | 2.00 | 2.00 | 1.00 | 3.00 | 3.00 | 2.50 | 1.50 | 2.00 | 1.00 |
| *C. hominis* & NTZ (n=5) | 0.50 | 1.50 | 1.00 | 1.50 | 1.00 | 1.00 | 1.50 | 0.75 | 1.75 | 1.00 | 1.00 |
|  | 1.00 | 3.00 | 1.50 | 2.50 | 3.00 | 4.00 | 3.75 | 4.00 | NA | NA | NA |
|  | 0.75 | 3.00 | 0.50 | 0.50 | 3.00 | 1.00 | 3.75 | 2.50 | 2.00 | 2.50 | 1.00 |
|  | 1.00 | 0.50 | 1.50 | 2.00 | 1.50 | 2.50 | 2.50 | 2.50 | 2.00 | 1.50 | 1.00 |
|  | 0.50 | 0.50 | 2.00 | 2.50 | 2.00 | 3.00 | 3.00 | 3.00 | 2.50 | 2.00 | 1.50 |
| *C. hominis* & AZR & NTZ (n=7) | 1.50 | 2.00 | 1.00 | 3.25 | 2.00 | 1.50 | 1.50 | 0.75 | 1.25 | 1.25 | 1.00 |
|  | 0.75 | 2.50 | 2.00 | 1.75 | 1.50 | 1.00 | 1.50 | 1.00 | 1.50 | 1.00 | 1.00 |
|  | 0.00 | 1.00 | 1.50 | 1.50 | 0.50 | 1.00 | 1.00 | 0.50 | 1.00 | 0.50 | 1.00 |
|  | 0.50 | 1.00 | 1.00 | 1.00 | 1.50 | 1.00 | 1.50 | 1.00 | 1.00 | 1.00 | 1.00 |
|  | 0.00 | 0.50 | 0.00 | 1.50 | 1.50 | 1.00 | 1.00 | 1.00 | 0.50 | 1.00 | 1.00 |
|  | 0.50 | 3.00 | 3.25 | 3.00 | 3.00 | 1.00 | 2.00 | 1.75 | 1.00 | 0.50 | 1.00 |
|  | 0.75 | 0.75 | 2.00 | 1.50 | 1.00 | 1.50 | 1.50 | 1.25 | 0.75 | 1.50 | 1.00 |
| AZR & NTZ (n=5) | 0.25 | 1.00 | 1.00 | 0.50 | 1.00 | 1.00 | 0.50 | 0.50 | 0.00 | 0.50 | 1.00 |
|  | 0.00 | 1.00 | 1.50 | 0.50 | 1.00 | 1.00 | 1.50 | 0.75 | 0.00 | 0.75 | 1.00 |
|  | 0.50 | 1.00 | 1.50 | 1.50 | 2.00 | 1.50 | 1.50 | 1.50 | 1.50 | 1.50 | 2.00 |
|  | 0.00 | 0.50 | 2.00 | 1.50 | 2.00 | 1.50 | 1.00 | 1.50 | 1.00 | NA | NA |
|  | 1.00 | 1.25 | 1.50 | 0.75 | 1.00 | 1.00 | 0.75 | 1.00 | 1.50 | 0.75 | 1.00 |
| None (n=4) | 0.50 | 0.00 | 0.25 | 0.50 | 0.00 | 0.00 | 0.50 | 0.25 | 0.50 | 0.00 | 0.00 |
|  | 0.00 | 0.50 | 0.50 | 1.00 | 1.00 | 1.00 | 0.50 | 1.00 | 1.00 | 0.00 | 0.50 |
|  | 0.00 | 0.50 | 0.50 | 1.00 | 0.50 | 0.50 | 0.50 | 1.00 | 1.00 | 1.00 | 0.50 |
|  | 1.00 | 1.50 | 1.50 | 1.50 | 1.50 | 0.75 | 0.25 | 1.00 | 1.00 | 0.75 | 1.00 |
